# Air pollution and corporate labor cost stickiness: Evidence from China

**DOI:** 10.1371/journal.pone.0335127

**Published:** 2025-10-24

**Authors:** Jiao Ma

**Affiliations:** School of Accounting, Southwestern University of Finance and Economics, Chengdu, Sichuan, China; University of Jaen: Universidad de Jaen, SPAIN

## Abstract

Air pollution is a common environmental issue worldwide, and its impact on macroeconomic development has always been of great concern. The role of air pollution on corporate behavior is a relatively new micro-perspective. By matching city-level air pollution data with data from A-share firms in China, the relationship between air pollution and corporate labor cost stickiness was examined. The study found that air pollution significantly increased corporate labor cost stickiness, especially for rank-and-file employees, with no significant impact on the labor cost stickiness of managers. Corporate good ESG performance can weaken the positive correlation between air pollution and labor cost stickiness. Air pollution exacerbates corporate labor cost stickiness through the mechanisms of salary compensation and the flow of labor forces. This positive correlation is more pronounced in firms located in areas with weaker traditional cultural influence, lower government focus on talent and lower environmental regulation stringency. This study enriches the research on the impact of air pollution on corporate governance, providing new evidence and ideas for the interdependence of environmental and economic benefits.

## Introduction

From 2013 to 2020, China’s air quality has significantly improved. At the same time, China faces the arduous task of industrial structure adjustment, the pollution sources in heavily polluted areas have not been completely eradicated, and the frequent occurrence of ozone weather, making air pollution control still a protracted battle. The China Ecological Environment Status Report shows that in 2018, the proportion of prefecture-level cities with air quality meeting standards was less than 40%, and by 2020, 40.1% of cities still did not meet air quality standards. In recent years, an increasing number of scholars have paid attention to the issue of air pollution, which can affect people’s physical and mental health, cognitive decision-making abilities, and mobility [1–3]. It also impacts the environmental and political costs of firms, their investment decisions, and innovation [4–6], and can influence the capital market as well as regional health levels and economic development [7]. Labor is the main body of a firm’s production and operations, and the impact of air pollution on firms can largely be realized through its effects on the workforce. However, there is a lack of academic research on the relationship between air pollution and labor costs.

After the reform and opening up, China’s economy has experienced rapid growth accompanied by a continuous rise in labor costs [8]. Although China’s labor costs are not as high as those in the United States and other countries, they are relatively high compared to Southeast Asia and other developing countries. The rise in labor costs has put forward new requirements for the country’s economic development direction, that is, the transformation and upgrading of the industrial structure, from general manufacturing to high-quality manufacturing [9,10]. At the same time, since labor cost decisions are a key part of a firm’s production and operation decisions, changes in labor costs also have a significant impact on firms. Existing studies have shown that corporate labor cost stickiness is influenced by factors such as adjustment costs [11], managerial internal factors [12], economic prospects [13], social characteristics [14], regulatory systems, company characteristics, and industry characteristics [15]. However, no studies have involved the impact of environmental factors such as ecological and environmental quality on corporate labor cost stickiness. Moreover, existing research on labor cost stickiness primarily focuses on the perspective of managers’ layoffs, lacking studies from the employees’ subjective viewpoint. We investigated whether air pollution affects corporate labor cost stickiness and analyzed the logic of the impact from the employees’ subjective perspective.

We take Chinese firms as our sample for three reasons. First, like other emerging markets, China has a large labor force, and research on labor cost stickiness in China can provide insights for other emerging markets. Second, the Labor Contract Law of the People’s Republic of China, implemented in 2008, has legally restricted corporate layoffs and strengthened the protection of workers, exacerbating corporate labor cost stickiness [16]. Legal protection has made the stickiness of labor costs a significant issue that Chinese firms cannot ignore. Third, over the past few decades, China has experienced rapid economic growth at the expense of the ecological environment, leading to air pollution issues. For instance, China has become the world’s largest source of carbon emissions. Studying the economic consequences of air pollution on firms within the Chinese context helps to better understand the interdependence between environmental and economic benefits.

We conducted our test using Chinese A-share listed firms from 2014 to 2020 as our sample. The study found that air pollution significantly increased corporate labor cost stickiness, and this effect remained after addressing endogeneity issues using instrumental variables and Difference-in-Differences (DID) methods, as well as after conducting other robustness tests. Air pollution increases the stickiness of labor costs for rank-and-file employees in firms, with no significant impact on the stickiness of labor costs for managers. Corporate good ESG performance can weaken the positive correlation between air pollution and labor cost stickiness. Air pollution exacerbates corporate labor cost stickiness through the mechanisms of salary compensation and the flow of labor forces. This positive correlation is more pronounced in firms located in areas with weaker traditional cultural influence, lower government focus on talent, lower environmental regulation stringency.

The main contributions of this paper are as follows: First, it includes air pollution as an ecological and environmental quality characteristic in the analysis of factors affecting corporate labor cost stickiness. Previous studies on the factors affecting corporate labor cost stickiness mainly involved internal and external systems and social culture, lacking consideration of ecological and environmental quality factors. This paper uses a large sample empirical test to examine the impact of air pollution, explore the mechanism, and deepen the understanding of corporate labor cost stickiness.

Second, it enriches the research on the economic consequences of air pollution. Studies on the economic consequences of air quality have not touched upon corporate labor cost stickiness. Some of the latest literature on air pollution and labor force has discussed the impact of air pollution on corporate labor strategies [17], mainly focusing on corporate labor employment decisions under air pollution, without considering the changes in labor costs when income changes under air pollution. Spatial economics research has shown that spatial characteristics such as weather and light can affect individuals’ judgment and decision-making [18]. This paper pioneers the study of the impact of air pollution on corporate labor cost stickiness, enriching the relevant research on the influence of air pollution on corporate governance.

Third, the research subjects have been refined. Existing literature has studied the impact of environmental regulation on labor demand across different industries [19]. Most of the literature takes corporate labor cost stickiness as a whole research object [11–15]. This paper further refines corporate labor cost stickiness into rank-and-file employees’ labor cost stickiness and managers’ labor cost stickiness. It aligns more closely with the logic of the active factors driving changes in labor costs and provides an explanation for labor cost changes driven by rank-and-file employees’ subjective factors.

## Literavture review and hypothesis development

### Literature review

#### Air pollution.

Existing studies have shown that air pollution can have an impact on individuals, firms, capital markets, and regions. First, air pollution can affect people’s physical and mental health, cognitive judgment ability, work, and production efficiency, including increasing the probability of respiratory diseases, leading to impaired cardiopulmonary function and the occurrence of diseases such as cancer, reducing life expectancy [20,21], cognitive ability and decision-making efficiency [1,2]; accumulating negative emotions, reducing mental health levels [22–25]; reducing individual working hours, lowering labor supply and work production efficiency [26–28], and prompting individuals to migrate [29–31]. Second, air pollution affects the political costs, investment decisions, preventive motives, innovative behaviors, and performance of firms. Specifically, air pollution leads to imposing extra political costs on firms in heavily polluted industries [7]; reducing corporate investment [4], increasing the risk of human capital loss [32], causing damage to corporate production efficiency and reduced performance [33,34]; making firms in areas with severe air pollution have preventive motives and aggressive earnings management decisions [35–37], affecting the strategic green innovation and overall innovation of firms [5,6]. Third, air pollution can affect the capital market by impacting the emotions and cognition of investors and analysts. Higher air quality can induce positive emotions in investors, reduce stock market volatility, and lead to higher returns, while lower air quality can increase the risk in the stock market [38], disrupt analysts’ judgment, and reduce the accuracy of their predictions [2].

#### Labor cost stickiness.

Traditional cost management theory posits a linear correlation between costs and business volume. However, an increasing number of studies have found that costs exhibit stickiness, meaning that the rate of increase in costs when business volume rises is greater than the rate of decrease in costs when business volume falls. Cost stickiness arises from managerial discretion in adjusting resources according to changes in demand [13]. It reflects the asymmetric changes in resource matching when business volume fluctuates. It also indicates a misalignment in corporate resource allocation. The factors influencing cost stickiness in existing research include three key drivers: adjustment costs [11], managerial optimism [12], and opportunistic management motives [39]. Labor cost stickiness refers to the phenomenon where the rate of increase in labor costs is greater when sales revenue increases than the rate of decrease in labor costs when sales revenue decreases.

Labor costs, as one kind of costs for firms, can have reasons for their stickiness explained by the aforementioned three factors. The impact of adjustment costs is characterized by the way management balances the costs and benefits of retaining unused resources versus the costs and benefits of adjusting them when business volume decreases. Adjustment costs include the tangible costs of layoffs, new hires, and training, as well as the intangible costs of reputation loss. The higher the adjustment costs, the lower the manager’s willingness to lay off employees and reduce production capacity [11,13]. The impact of managerial optimism is manifested by the fact that estimates of future business volume depend on the subjective judgment of management. More optimistic management is more likely to overestimate future business volume. They may view a decline in business as temporary. Thus, they avoid the additional costs associated with layoffs and subsequent recovery of production capacity [12]. Opportunistic management motives stem from agency costs. In pursuit of personal utility such as status and material benefits brought about by empire building, management tends to expand the firm beyond the optimal size. Management is reluctant to make decisions to reduce idle resources when business volume declines, such as layoffs [14]. Factors such as managerial incentives [40,41], economic prospects [42], social characteristics [14], legal and regulatory systems [16], company characteristics, and industry characteristics [15,43–45] can affect cost stickiness. For example, driven by the motivation to reach profitability goals, self-interested management may be inclined to cut idle resources when business volume decreases to avoid losses, thereby reducing the degree of cost stickiness [40,41]. Previous research has focused on analyzing the causes and changes of labor cost stickiness from the perspective of managers, lacking analysis at the subjective level of general employees.

#### Air pollution and labor cost stickiness.

To date, no literature has explored the impact of air pollution on corporate labor cost stickiness. Relevant studies have focused on the effects of air pollution on labor mobility, employee welfare, and labor costs. Empirical research using Chinese provincial panel data indicates that air pollution prompts labor outflow, with a weaker correlation observed in economically developed provinces [46]. A positive correlation exists between air pollution and the willingness of labor mobility, as evidenced by the increased frequency of searches for “immigration” on search engines due to air pollution [47]. Wang et al. (2021) [32] measured employee treatment by the income and care scores given to firms by employees of Chinese-listed manufacturing firms. Their study found that under air pollution, firms improve employee treatment to motivate employees and reduce the costs associated with rehiring talent. Research using data from Chinese industrial firms shows that air pollution increases corporate health expenditures for employees, reduces labor supply, and is positively correlated with labor costs [48]. These prior studies have laid a preliminary foundation for understanding the impact of air pollution on regional labor mobility and corporate employee welfare. However, the literature mentioned above has not been conducted using a large sample of Chinese listed firms, nor has it differentiated the impact on corporate management and rank-and-file employees. Studies on labor costs have not focused on the relative changes in labor costs when corporate sales revenue rises and falls under air pollution, that is, the changes in labor cost stickiness. Labor mobility and employee treatment are important components that affect labor cost stickiness and may have a close relationship with each other. Investigating the impact of air pollution on corporate labor cost stickiness can provide a new perspective for better understanding the behavior of firms and employees under air pollution and possible countermeasures.

### Hypothesis development

The existing literature on labor cost stickiness focuses on explanations from the perspective of corporate decision-making, but the subjective choices of the workforce cannot be ignored. Clean air is a non-monetary benefit for employees [3]. When air pollution occurs, rational employees need to weigh good air quality against monetary income. They either stay in the polluted area and demand compensation or move to a place with better air quality. Air pollution, as a phenomenon closely related to human health and well-being, affects the willingness of workers to stay in their current location [29–31,49]. The expected benefits for employees mainly come from both the firm and the local area. When air pollution occurs, firms may compensate for employees’ health losses by increasing their monetary income through salary increases. This reduces their willingness to move elsewhere, minimizes the risk of human capital loss, and thus increases corporate labor cost stickiness. The benefits provided by the local area can substitute for those provided by firms. These include non-material benefits such as culture [50], policy-related benefits such as healthcare, education, and talent care [51,52], and monetary benefits such as talent subsidies. When all these benefits meet the employees’ expectations, they will stay with the firm.

The existing literature on labor cost stickiness lacks a detailed analysis of its impact on managers and rank-and-file employees. The impact of air pollution on the labor cost stickiness of managers and rank-and-file employees is different. This difference stems from the different compensation incentive structures of rank-and-file employees and managers. Managers have a higher bargaining power in the human capital market [53], and the existence of information asymmetry makes the compensation contracts of managers closely related to performance [54]. For managers, the environment of the firm’s location has little impact on them, because there is no obvious relationship between the environment of the firm’s location and their compensation. The main factor determining which firm managers choose to work for is performance. When a firm’s income decreases, the firm is unlikely to provide additional compensation to managers based on the compensation contract. Therefore, managers are less sensitive to air pollution, and air pollution may have no impact on the labor cost stickiness of managers. The compensation of rank-and-file employees is closely related to their positions and has no relation to the firm’s performance. When the positions in different places are the same, rank-and-file employees need to consider the market-level compensation. They also need to consider environmental factors, such as air pollution. Therefore, the additional compensation provided by the firm and the spiritual and material benefits provided by the local area are more important. Thus, air pollution is likely to affect the labor cost stickiness of rank-and-file employees.

Air pollution may affect corporate labor cost stickiness through the following mechanisms.

First, air pollution may prompt firms to provide salary compensation to the labor force, increasing the stickiness of labor costs. A key factor in determining the level of employees’ sense of belonging to a firm is job satisfaction [55], and the compensation and promotion opportunities obtained in the workplace are likely to affect job satisfaction. Employees working in firms located in areas with severe air pollution perceive the risks associated with air pollution, incurring additional health costs such as medical expenses [56]. In addition to the consideration of their health, employees may also take leave to care for sick family members [57], which incurs higher costs for them. When the benefits employees receive are relatively lower compared to the costs they bear, their work enthusiasm will decline. When the costs associated with air pollution exceed the existing benefits of employees, they will demand higher benefits [48], which include benefits from both the firm and the local area. When air pollution is severe, if firms want to maintain employees’ work enthusiasm and efficiency, they need to provide additional compensation to cover the medical costs of employees [58]. Therefore, firms in areas with severe air pollution may offer salary compensation to employees, thereby increasing labor cost stickiness.

Second, air pollution may change the mobility of the labor force, increasing corporate labor cost stickiness. Research in neoclassical economics shows that income and the cost of migration are important variables affecting population migration [59,60]. In addition, formal institutions and informal institutions may also affect the willingness of population migration [61]. The process of labor migration is a process of seeking places with higher marginal output [62]. Rational individuals will act to obtain the highest personal benefits [59,63]. Air pollution is an adverse natural environmental characteristic. Air pollution reduces the quality of life of employees. When the net present value of migration is greater than zero, it will trigger the mobility of employees. Employees in areas with air pollution are subjectively willing to migrate [47]. When air pollution is severe, firms face a higher risk of talent loss compared to non-polluted situations. To prevent the loss of human capital, firms in areas with poor air quality are likely to increase employees’ income or offer them more promotion opportunities, making the benefits of staying greater than the costs, thereby leading them to decide to stay with the original firm. On the other hand, when the treatment offered by firms is attractive enough, it may attract employees from other firms, resulting in a talent inflow effect.. Therefore, firms in areas with severe air pollution may take compensatory measures to retain and attract employees, increasing corporate labor cost stickiness. Based on the above analysis, the hypotheses are as follows.

H1. Air pollution can exacerbate corporate labor cost stickiness.

H2. Salary compensation is a mediator between air pollution and corporate labor cost stickiness.

H3. The flow of labor forces is a mediator between air pollution and corporate labor cost stickiness.

In 2006, the United Nations Principles for Responsible Investment (UN-PRI) first introduced the ESG (Environmental, Social and Governance) concept, and encouraged and promoted member institutions to incorporate ESG factors into corporate operations. ESG assesses the sustainability of corporate operations from three dimensions: environment, society and corporate governance. Through the ESG scores of firms, investors can evaluate the performance of firms in terms of sustainable operations, environmental responsibility and social responsibility fulfillment. Good ESG performance affects the positive correlation between air pollution and corporate labor cost stickiness. From the perspective of organizational identification, firms with good ESG performance can gain a better reputation. An enhanced corporate reputation strengthens employees’ organizational identification, leading to a stronger sense of belonging [64]. This sense of belonging is a non-material benefit that employees value in their work, prompting them to act in line with the organization’s interests. When facing a decline in revenue, firms with good ESG performance in areas with severe air pollution may find that employees are willing to accept lower compensation. This willingness is due to the non-material benefits of reputation and organizational identification. Therefore, firms with good ESG performance can reduce the strength of the positive correlation between air pollution and labor cost stickiness by diminishing the need for high compensation.

In addition, firms with good ESG performance can obtain more financing support. According to signaling theory, there is information asymmetry between the firm’s internal operations and its stakeholders. When a firm actively takes on social or environmental responsibilities, it can send positive signals to its stakeholders [65]. After receiving these positive signals, stakeholders will increase their trust in the firm and provide corresponding positive feedback. For example, the firm will more easily gain the trust of investors, and financial institutions and governments will provide more favorable financing conditions for the firm. As environmental protection and green development are increasingly valued by government departments, firms that actively take on environmental responsibilities can reduce their debt financing costs [66]. In areas with severe air pollution, firms with good ESG performance may have a greater capacity and willingness to increase spending on environmental protection and employee benefits, which may strengthen the positive correlation between air pollution and labor cost stickiness.

Based on the above analysis, the hypothesis is as follows.

H4a. The impact of air pollution on corporate labor cost stickiness is weaker in firms with better ESG performance.

H4b. The impact of air pollution on corporate labor cost stickiness is stronger in firms with better ESG performance.

## Methodology

### Data

This paper takes the listed firms in China from 2014 to 2020 as the sample. Before 2013 in China, the Air Pollution Index (API) was used to denote the degree of urban air pollution. After 2013, in accordance with the requirements of the newly issued Environmental Air Quality Standards, the Air Quality Index (AQI) was used as a substitute, and monitoring data was collected. However, since 2013 was the first year of pilot monitoring, data for most cities were missing, so this paper chooses 2014 as the starting year for sample selection. In 2021, China achieved the national coordination of the basic pension insurance system, which increased the cost burden on firms for employing workers. Using data from and after 2021 could impact the research findings, hence this paper selects 2020 as the terminal year for sample selection. The measurement of urban air pollution in this paper is based on the original data from the national urban air quality daily report data of the cities where firms are located in the CNRDS database. Since the empirical analysis is based on annual data, the average value of the daily urban air quality data is used as the annual data. Urban inversion data comes from the NASA MERRA2 satellite dataset, which is calculated based on the temperature at 42 global pressure levels. Urban population data comes from the “China Urban Statistical Yearbook” and the population census data bulletin.

Corporate data comes from the Wind and CSMAR Economic and Financial Research Database, and the Huazheng ESG Ratings are used to denote the firm’s ESG performance. Huazheng ESG Ratings comprehensively considers the performance of A-share listed firms in China in terms of environmental, social, and corporate governance aspects and provides corresponding rating scores. Overall, this index has three advantages compared to other domestic ESG rating data: it is more adapted to the characteristics of the Chinese market, has a wide coverage, and is timely and prompt. By merging AQI data, inversion data, and corporate data based on the city where a firm is located. This paper excludes samples with key data missing, financial industries, and samples with abnormal financial conditions, and finally organizes 5288 listed firm-annual observations. All continuous variables in this paper have been winsorized at the 1% level on both sides.

### Model design and variables

#### Air pollution and corporate labor cost stickiness.

Following the approach of Anderson et al. (2003) [13], the study first tests for the presence of corporate labor cost stickiness.


$$\Delta Lcost_{it}=\alpha_{0}+\alpha_{1}\Delta Rev_{it}+\alpha_{2}De_{it}*\Delta Rev_{it}+Firm_{i}+Year_{t}+\varepsilon it$$ (1)

ΔLcost is the change in the natural logarithm of the total labor cost of firm i in year t. ΔRev is the change in the natural logarithm of sales revenue of firm i in year t. De is a dummy variable indicating whether the firm’s sales revenue has decreased in year t, with 1 indicating a decrease and 0 otherwise. α_1_ denotes the percentage increase in labor costs when sales revenue increases by 1%; α_1_ + α_2_ is the percentage decrease in labor costs when sales revenue decreases by 1%. If α_2_ is less than 0, it indicates that the firm has labor cost stickiness.

The core of this study is to investigate the impact of air pollution on corporate labor cost stickiness, that is, the impact on α_2_ in model (1). Following the approach of Liu and Liu (2014) [16] and Xu et al. (2023) [43], a model is constructed for the influencing factors of α_2_.


$$\begin{array}{c} \alpha_{2}=\beta_{0}+\beta_{1}AQI_{it}+\beta_{2}AI_{it}+\beta_{3}LI_{it}+\beta_{4}Lev_{it}+\beta_{5}GDPg_{it}+\beta_{6}SucDe_{it}\\ +\beta_{7}ESGH_{it}+\beta_{8}ESGH_{it}*AQI_{it} \end{array}$$ (2)

AQI refers to the annual average of the daily AQI in the city where firm i is located, and ESGH is a dummy variable that takes the value of 1 if a firm’s ESG score for the year is above the median score of all firms, and 0 otherwise. Following the research of Anderson et al (2003) [13], Dierynck et al (2012) [41], and Xu et al (2023) [43], the control variables include: labor intensity (LI), asset intensity (AI), financial leverage (Lev), GDP growth rate of the province where a firm is located (GDPg), and consecutive two-year sales decline (SucDe), with specific variable definitions provided in Table 1. The study further controls for fixed effects at both the firm and annual levels.

**Table 1:** Main variable definition.

Variable	Symbol	Variable construction	Data source
The change in the natural logarithm of labor cost	**ΔLcost**	*ΔLcost*_*it*_* = *ln(*Lcost*_*it*_)*-*ln(*Lcost*_*i,t-1*_); the labor cost (*Lcost*) equals the “increase in employee compensation payable” on the balance sheet at the end of the year.	CSMAR
The change in the natural logarithm of the total labor cost for rank-and-file employees	**ΔLcost_staff**	*ΔLcost_staff*_*it*_* = *ln(*Lcost*_*_*_*staff*_*it*_)*-*ln(*Lcost*_*_*_*staff*_*i,t-1*_);the labor cost for rank-and-file employees (*Lcost*_*_*_*staff*) is equal to the “increase in employee compensation payable” on the balance sheet at the end of the year minus the employee compensation in the “administrative expenses” item in the notes to the financial statements.	CSMAR
The change in the natural logarithm of the total labor cost for managers	**ΔLcost_exe**	*ΔLcost_exe*_*it*_* = *ln(*Lcost*_*_*_*exe*_*it*_)*-*ln(*Lcost*_*_*_*exe*_*i,t-1*_); the labor cost for managers (*Lcost*_*_*_*exe*) is equal to the employee compensation in the “administrative expenses” item in the notes to the financial statements.	CSMAR
Air quality	**AQI**	The annual average of the daily AQI in the city where a firm is located	CNRDS
The change in the natural logarithm of sales revenue	**ΔRev**	*ΔRev*_*it*_* = *ln(*Rev*_*it*_)*-*ln(*Rev*_*i,t-1*_)	CSMAR
Decrease in sales revenue	**De**	The value is 1 if *Rev*_*i,t-1*_* > Rev*_*it*_ and 0 otherwise	CSMAR
Labor intensity	**LI**	ln(total number of employees/ operating income *100000)	CSMAR
Asset intensity	**AI**	ln(total assets/ operating income)	CSMAR
Financial leverage	**Lev**	Total liabilities to total assets ratio	CSMAR
GDP growth rate	**GDPg**	The ratio of GDP growth by province	Hand-collected
Consecutive two-year decline in sales revenue	**SucDe**	The value is 1 if *Rev*_*i,t-2*_* > Rev*_*i,t-1*_* > Rev*_*it*_ and 0 otherwise	CSMAR
Good ESG performance	**ESGH**	The value is 1 if a firm’s ESG score for the year is higher than the median score of all firms and 0 otherwise	Huazheng ESG Ratings
The change in the natural logarithm of per capita compensation for employees	**ΔEpay**	Δ*Epay = *ln(*Epay*_*it*_)*-*ln(*Epay*_*i,t-1*_)	CSMAR

We substitute model (2) into model (1) to obtain model (3), and further control for firm and year fixed effects.


$$\begin{array}{c} \Delta Lcost_{it}=\alpha_{0}+\alpha_{1}\Delta Rev_{it}+(\beta_{0}+\beta_{1}AQI_{it}+\beta_{2}AI_{it}+\beta_{3}LI_{it}+\beta_{4}Lev_{it}+\beta_{5}GDPg_{it}+\beta_{6}SucDe_{it}\\ +\beta_{7}ESGH_{it}+\beta_{8}ESGH_{it}*AQI_{it})*De_{it}*\Delta Rev_{it}+Firm_{i}+Year_{t}+\varepsilon it \end{array}$$ (3)

In model (3), if β_1_ is negative, it indicates that air pollution exacerbates corporate labor cost stickiness. If β_8_ is positive, it suggests that a better ESG performance can mitigate the positive correlation between air pollution and corporate labor cost stickiness.

To study the impact of air pollution on the labor cost stickiness of rank-and-file employees and managers, models (4) and (5) are constructed:


$$\begin{array}{c} \Delta Lcost\_staff_{it}=\alpha_{0}+\alpha_{1}\Delta Rev_{it}+(\beta_{0}+\beta_{1}AQI_{it}+\beta_{2}AI_{it}+\beta_{3}LI_{it}+\beta_{4}Lev_{it}+\beta_{5}GDPg_{it}\\ +\beta_{6}SucDe_{it}+\beta_{7}ESGH_{it}+\beta_{8}ESGH_{it}*AQI_{it})*De_{it}*\Delta Rev_{it}+Firm_{i}+Year_{t}+\varepsilon it \end{array}$$ (4)

$$\begin{array}{c} \Delta Lcost\_exe_{it}=\alpha_{0}+\alpha_{1}\Delta Rev_{it}+(\beta_{0}+\beta_{1}AQI_{it}+\beta_{2}AI_{it}+\beta_{3}LI_{it}+\beta_{4}Lev_{it}+\beta_{5}GDPg_{it}\\ +\beta_{6}SucDe_{it}+\beta_{7}ESGH_{it}+\beta_{8}ESGH_{it}*AQI_{it})*De_{it}*\Delta Rev_{it}+Firm_{i}+Year_{t}+\varepsilon it \end{array}$$ (5)

ΔLcost_staff_it_ is the change in the natural logarithm of the total labor cost for rank-and-file employees. ΔLcost_exe_it_ is the change in the natural logarithm of the total labor cost for managers.

The definitions of the remaining variables are consistent with those in model (3).

#### Testing from salary compensation.

Drawing on the approaches of Dierynck et al (2012) [41] and Xu et al (2023) [43], the following model is established to test whether the impact of air pollution on corporate labor cost stickiness is based on changes in salary compensation.


$$\begin{array}{c} \Delta Epay_{it}=\alpha_{0}+\alpha_{1}\Delta Rev_{it}+(\beta_{0}+\beta_{1}AQI_{it}+\beta_{2}AI_{it}+\beta_{3}LI_{it}+\beta_{4}Lev_{it}+\beta_{5}GDPg_{it}+\beta_{6}SucDe_{it}\\ +\beta_{7}ESGH_{it}+\beta_{8}ESGH_{it}*AQI_{it})*De_{it}*\Delta Rev_{it}+Firm_{i}+Year_{t}+\varepsilon it \end{array}$$ (6)

In model (6), the dependent variable ΔEpay refers to the change in the natural logarithm of per capita compensation for employees. If the sign of β_1_ is consistent with the sign of β_1_ in model (3), then the impact of air pollution on corporate labor cost stickiness is based on changes in salary compensation.

#### Testing from the flow of labor forces.

Referencing previous literature [67], we use the net inflow of population in the city where a firm is located to measure the personnel mobility of the firm. If the net population inflow of the city where the firm is located in the current year is higher than the median of all cities, it is classified as the inflow group (Inflow = 1). Otherwise, it is classified as the outflow group (Inflow = 0). A Group test is conducted on model (3).

## Results

### Descriptive statistics

Table 2 presents the descriptive statistical results of the main variables. The average value of ΔLcost is 0.1113, indicating an upward trend in labor costs for the sample firms, with the maximum value being 4.3675 and the minimum value being −3.3010. The average value of ΔRev is 0.0892, which is less than the average value of ΔLcost, with the maximum value being 4.0858 and the minimum value being −2.5052. The mean of De is 0.3028, suggesting that 30.28% of the sample firms have experienced a decline in sales revenue. The mean of SucDe is 0.1225, indicating that 12.25% of the sample companies have seen a continuous two-year decline in sales revenue. The aforementioned values are generally consistent with existing literature.

**Table 2:** Descriptive statistics.

Variable	N	Mean	Med	Sd	Min	Max
**ΔLcost**	5288	0.1113	0.0864	0.2778	−3.3010	4.3675
**ΔLcost_staff**	5288	0.1111	0.0871	0.3687	−5.5178	5.7630
**ΔLcost_exe**	5288	0.1095	0.0914	0.3038	−2.3479	4.9769
**AQI**	5288	72.7174	70.7568	21.8406	29.2923	178.0526
**ΔRev**	5288	0.0892	0.0797	0.3199	−2.5052	4.0858
**De**	5288	0.3028	0.0000	0.4595	0.0000	1.0000
**LI**	5288	−2.5688	−2.4718	0.8946	−6.8710	0.5277
**AI**	5288	0.7505	0.6901	0.7258	−2.1519	4.8635
**Lev**	5288	0.4799	0.4877	0.2006	0.0777	0.9077
**GDPg**	5288	0.1326	0.0829	0.3471	−0.5393	2.0547
**SucDe**	5288	0.1225	0.0000	0.3279	0.0000	1.0000
**ESGH**	5288	0.5306	1.0000	0.4991	0.0000	1.0000
**ΔEpay**	5288	0.0721	0.0683	0.2222	−0.7415	1.0638

### Base regression results

Table 3 presents the basic regression results. Column (1) reflects the results of model (1), which indicates whether the sample firms have labor cost stickiness. The regression coefficient of ΔRev is 0.6524, significantly positive (p = 0.0001, p < 0.01), indicating that when sales revenue increases by 1%, labor costs increase by 0.6524%. The regression coefficient of De*ΔRev is −0.4990, significantly negative (p = 0.0001, p < 0.01), indicating that when sales revenue decreases by 1%, labor costs decrease by 0.1534% (0.6524%−0.4990%). This indicates that the sample firms have labor cost stickiness, which is consistent with the results of existing literature and the expectations of this paper. The model in column (2) adds De *ΔRev*AQI to the model in column (1) for regression. The coefficient of De *ΔRev*AQI is −0.0016, significantly negative (p = 0.0001, p < 0.1), indicating that, on average, the more severe the air pollution in the city where a firm is located, the higher the average labor cost stickiness. When sales decrease by 1%, and the annual average value of the AQI index in the city where the firm is located increases by one unit, the decline in the firm’s labor cost will be lessened by 0.0016%.

**Table 3:** Air pollution and corporate labor cost stickiness.

Variable	ΔLcost			△Lcost_staff	△Lcost_exe
	(1)	(2)	(3)	(4)	(5)
**ΔRev**	0.6524***	0.6537***	0.6591***	0.6529***	0.5274***
	(45.1934)	(45.2396)	(46.3061)	(30.4955)	(29.5705)
De *ΔRev	−0.4990***	−0.3913***	−0.5663***	−0.5413***	−0.2290
	(−16.9864)	(−5.9396)	(−4.7861)	(−3.0413)	(−1.5446)
De *ΔRev*AQI		−0.0016*	−0.0031**	−0.0051***	−0.0006
		(−1.8261)	(−2.5478)	(−2.7909)	(−0.4081)
De *ΔRev* LI			−0.1756***	−0.3058***	−0.0934***
			(−8.2155)	(−9.5141)	(−3.4864)
De *ΔRev* AI			0.0107	0.0997***	−0.0698***
			(0.5837)	(3.6153)	(−3.0361)
De *ΔRev* Lev			−0.4294***	−0.8012***	−0.4628***
			(−5.3977)	(−6.6951)	(−4.6429)
De *ΔRev* GDPg			−8.2782*	−16.4342**	−9.4201
			(−1.6476)	(−2.1747)	(−1.4963)
De *ΔRev* SucDe			0.3472***	0.4485***	0.0688
			(9.1374)	(7.8477)	(1.4452)
De *ΔRev* ESGH			−0.4202***	−0.5402***	−0.2389
			(−3.3828)	(−2.8913)	(−1.5352)
De *ΔRev* ESGH* AQI			0.0034*	0.0044*	0.0028
			(1.9541)	(1.6580)	(1.2541)
**Constant**	0.0714***	0.0692***	0.0663***	0.0792***	0.0848***
	(7.5188)	(7.2262)	(7.0175)	(5.5774)	(7.1629)
**Firm/Year**	Yes	Yes	Yes	Yes	Yes
**N**	5288	5288	5288	5288	5288
**Within-R** ^**2**^	0.3881	0.3886	0.4136	0.2718	0.2266

Note: T-statistics are reported in parentheses. ***, **, and * indicate regression coefficients significant 1%, 5%, and 10%, respectively.

Model (3) specifically tests the relationship between air pollution and corporate labor cost stickiness. In column (3), the coefficient of De*ΔRev*AQI is −0.0031, significantly negative (p = 0.011, p < 0.05), indicating that when sales decrease by 1%, for every one-unit increase in the annual average AQI index of the city where the firm is located, the decline in the firm’s labor cost will be reduced by 0.0031%. And its absolute value is greater than the absolute value of the coefficient of De *ΔRev*AQI in column (2), indicating that after incorporating other factors that affect corporate labor cost stickiness, air pollution more significantly increases corporate labor cost stickiness, thus supporting H1. The coefficient of De *ΔRev* ESGH* AQI is 0.0034, significantly positive (p = 0.051, p < 0.1), indicating that good ESG performance can weaken the positive correlation between air pollution and corporate labor cost stickiness, thus supporting H4a. The coefficient of De*ΔRev*AQI in column (4) is −0.0051, significantly negative (p = 0.005, p < 0.01), indicating that when sales decrease by 1%, for every one-unit increase in the annual average AQI index of the city where the firm is located, the decline in the labor cost of the firm’s ordinary employees will be reduced by 0.0051%. The coefficient of De *ΔRev*AQI in column (5) is not significant. The results demonstrate that air pollution will exacerbate the labor cost stickiness of rank-and-file employees, but has no significant impact on the labor cost stickiness of managers. Air pollution has no significant impact on the stickiness of labor costs for managers. This is mainly because management compensation is closely related to performance. There is no significant link between the environmental quality of a firm’s location and management compensation. When corporate revenue declines, the firm is unlikely to provide economic compensation to managers based on compensation contracts.

## Mechanism test

Based on the theoretical analysis in the previous text, first, air pollution may intensify labor cost stickiness by enhancing salary compensation. Since the main regression shows that air pollution will exacerbate the labor cost stickiness of rank-and-file employees, model (6) sets ΔEpay as the change in the natural logarithm of the average compensation of rank-and-file employees. The result in column (1) of Table 4 shows that the estimated coefficient of De*ΔRev*AQI is significantly negative (p = 0.094, p < 0.1), and the estimated coefficient of De*ΔRev* ESGH* AQI is significantly positive (p = 0.034, p < 0.05). This indicates that when sales decrease by 1%, for every one-unit increase in the annual average AQI index of the city where the firm is located, the decline in the average salary of ordinary employees in the firm will be reduced by 0.0022%. Air pollution prompts firms to provide salary compensation to ordinary employees, increasing labor cost stickiness, thus supporting H2. At the same time, firms with good ESG performance can reduce the intensity of compensation, thereby weakening the positive correlation between air pollution and corporate labor cost stickiness. Additionally, air pollution may affect labor cost stickiness by altering the flow of labor forces. The regression results of the group tests in columns (2) and (3) of Table 4 show that in the inflow group (Inflow = 1), the estimated coefficient of De*ΔRev*AQI is not significant. In the outflow group (Inflow = 0), the estimated coefficient of De*ΔRev*AQI is −0.0071, which is significantly negative (p = 0.001, p < 0.01). The difference in coefficients between the two groups is significant, indicating that air pollution prompts firms to retain employees, thereby increasing labor cost stickiness. Thus, H3 is supported.

**Table 4:** Mechanism test.

Variable	ΔEPay	ΔLcost	
	(1)	Inflow = 1	Inflow = 0
		(2)	(3)
**ΔRev**	0.1394***	0.5983***	0.6249***
	(9.0105)	(27.0017)	(19.2220)
De *ΔRev	0.1343	−1.0098***	0.0757
	(1.0440)	(−5.4889)	(0.2317)
De *ΔRev*AQI	−0.0022*	−0.0012	−0.0071***
	(−1.6758)	(−1.2925)	(−3.2170)
De *ΔRev* LI	0.0082	−0.3860***	−0.0199
	(0.3544)	(−11.4967)	(−0.3156)
De *ΔRev* AI	0.0243	0.0803**	−0.0397
	(1.2175)	(2.1501)	(−0.9572)
De *ΔRev* Lev	−0.0304	−0.5324***	−0.3594**
	(−0.3520)	(−4.1581)	(−2.2229)
De *ΔRev* GDPg	−17.9252***	−15.0767*	30.3484***
	(−3.2828)	(−1.8490)	(2.7788)
De *ΔRev* SucDe	0.0569	0.5731***	0.1227
	(1.3779)	(9.3055)	(1.1891)
De *ΔRev* ESGH	−0.2336*	−0.5803***	−0.7676***
	(−1.7303)	(−2.6625)	(−3.4715)
De *ΔRev* ESGH* AQI	0.0041**	0.0042	0.0089***
	(2.1185)	(1.3578)	(2.8752)
**Constant**	0.0847***	0.0776***	0.0590**
	(8.2521)	(5.2172)	(2.3218)
**Firm/Year**	Yes	Yes	Yes
**N**	5288	2145	916
**Within-R** ^**2**^	0.0585	0.4476	0.4381
**p-value**		0.05	

Note: T-statistics are reported in parentheses. ***, **, and * indicate regression coefficients significant 1%, 5%, and 10%, respectively. △*EPay* refers to the change in the natural logarithm of the average compensation of rank-and-file employees. If the net population inflow of the city where the firm is located in the current year is higher than the median of all cities, it is classified as the inflow group (*Inflow* = 1). Otherwise, it is classified as the outflow group (*Inflow* = 0).

### Heterogeneity analysis

#### Considering traditional culture.

Formal and informal institutions may affect people’s satisfaction with their place of residence [68]. Culture, as an informal institution, can influence individuals’ subjective preferences, and thus affect their action decisions [61,69]. Traditional cultures such as clan culture can form a close emotional connection between employees and firms, which is a kind of regional spiritual benefit for employees [50]. In areas where traditional culture has a profound influence, people have a higher sense of identification with the collective, and have a high level of enthusiasm for work and may not require substantial wage compensation when facing air pollution. Therefore, traditional culture may have a substitutable relationship with the salary compensation of firms. This paper speculates that the exacerbating effect of air pollution on corporate labor cost stickiness is more pronounced in firms located in areas where the influence of traditional culture is weaker. Referring to the measurement method of clan culture by Greif and Tabellini [45], if the number of genealogy volumes per million people in the province where a firm is located is higher than the median of all provinces, the firm is classified as a strong traditional culture group (Culture = 1); otherwise, it is classified as a weak traditional culture group (Culture = 0). Columns (1) and (2) of Table 5 show the empirical test results. It can be seen that in the strong traditional culture group (Culture = 1), the coefficient of De*ΔRev*AQI is not significant, while in the weak traditional culture group (Culture = 0), the coefficient of De*ΔRev*AQI is significantly negative (p = 0.0001, p < 0.01). This indicates that when the influence of traditional culture is strong, firms in regions with severe air pollution do not need to offer employees a large amount of salary compensation to retain them. The results demonstrate that the positive correlation between air pollution and corporate labor cost stickiness is more pronounced in firms located in areas with weaker traditional cultural influence.

**Table 5:** Heterogeneity analysis.

Variable	ΔLcost					
	Culture = 1	Culture = 0	Tpolicy = 1	Tpolicy = 0	Epolicy = 1	Epolicy = 0
	(1)	(2)	(3)	(4)	(5)	(6)
**ΔRev**	0.6837***	0.6248***	0.7806***	0.5373***	0.6524***	0.6252***
	(35.2266)	(30.1552)	(36.5044)	(26.2212)	(31.4084)	(27.4712)
De *ΔRev	−0.6336***	−0.5852***	−0.2831	−0.9281***	−1.3322***	0.3574*
	(−3.5110)	(−3.5803)	(−1.1820)	(−5.6444)	(−7.7497)	(1.6872)
De *ΔRev*AQI	0.0003	−0.0060***	−0.0014	−0.0032**	−0.0021	−0.0088***
	(0.1576)	(−3.2377)	(−0.4658)	(−2.1598)	(−1.3157)	(−3.4265)
De *ΔRev* LI	−0.0383	−0.2898***	0.0633*	−0.3433***	−0.3667***	−0.0107
	(−1.1704)	(−9.7699)	(1.7548)	(−11.1386)	(−11.0455)	(−0.3213)
De *ΔRev* AI	−0.0464**	0.1195***	−0.0571	0.1036***	0.0929***	−0.1015***
	(−1.9644)	(3.2883)	(−1.5995)	(4.0612)	(3.5845)	(−2.9985)
De *ΔRev* Lev	0.1507	−0.7877***	0.1586	−0.6329***	−0.7603***	−0.0029
	(1.2052)	(−7.1621)	(1.0765)	(−5.8801)	(−6.5518)	(−0.0222)
De*ΔRev* GDPg	−7.6757	−12.8296**	−16.4127**	8.4764	17.6502**	−10.0228
	(−0.9380)	(−1.9754)	(−2.1342)	(1.1447)	(2.1337)	(−1.3488)
De*ΔRev* SucDe	0.1520***	0.4787***	0.1335**	0.3830***	0.5503***	0.0813
	(2.8110)	(8.7325)	(2.0415)	(6.8922)	(9.6696)	(1.3211)
De*ΔRev* ESGH	−0.0992	−0.3186	−0.0318	−0.7685***	−0.4985***	−0.6842***
	(−0.5291)	(−1.6352)	(−0.1302)	(−4.6097)	(−2.9074)	(−2.8778)
De*ΔRev*ESGH* AQI	−0.0011	0.0034	−0.0013	0.0080***	0.0067***	0.0074**
	(−0.3669)	(1.3352)	(−0.3733)	(3.4504)	(2.7542)	(2.2456)
**Constant**	0.0773***	0.0519***	0.0624***	0.0662***	0.0700***	0.0689***
	(5.6271)	(4.0490)	(4.5795)	(4.5704)	(4.8445)	(5.1193)
**Firm/Year**	Yes	Yes	Yes	Yes	Yes	Yes
**N**	2595	2693	2583	2705	2607	2681
**Within-R** ^**2**^	0.4340	0.4184	0.4610	0.4002	0.4485	0.3594
**p-value**	0.05		0.05		0.05	

Note: T-statistics are reported in parentheses. ***, **, and * indicate regression coefficients significant 1%, 5%, and 10%, respectively. If the number of genealogy volumes per million people in the province where a firm is located is higher than the median of all provinces, the firm is classified as a strong traditional culture group (*Culture* = 1); otherwise, it is classified as a weak traditional culture group (*Culture* = 0). If the talent attention of the city where a firm is located is higher than the median of all cities in that year, the firm is classified as the high talent focus group (*Tpolicy* = 1), otherwise it is classified as the low talent focus group (*Tpolicy* = 0). If the environmental regulation intensity in the city where a firm is located (the proportion of the total investment in urban industrial pollution control to the industrial added value) is higher than the median of all cities in that year, the firm is classified as the strong environmental regulation group (*Epolicy* = 1); otherwise, it is classified as the weak environmental regulation group (*Epolicy* = 0).

#### Considering the government’s focus on talent development.

The theoretical analysis section mentions that employees’ expected benefits include potential non-monetary benefits such as healthcare, education, and talent care [51,52]. Whether the city where the firm is located is favorable to talent is a key factor in influencing talent’s identification with the city [70,71]. The allocation of attention can be understood as an indication of the government’s resource allocation tendencies in the foreseeable future. The higher the level of government attention, the more financial and human resources are likely to be allocated to a particular field. The higher the government’s focus on talent, the more likely it is to increase non-monetary benefits for talent, such as healthcare and education. When employees receive additional compensation from the government, their demand for compensation from firms will decrease. Therefore, this paper speculates that the exacerbating effect of air pollution on corporate labor cost stickiness is more pronounced in firms in areas with low government talent attention. Based on the frequency of keywords related to talent in the work reports of prefecture-level city governments, a talent focus index is constructed. If the talent attention of the city where a firm is located is higher than the median of all cities in that year, the firm is classified as the high talent focus group (Tpolicy = 1), otherwise, it is classified as the low talent focus group (Tpolicy = 0). Columns (1) and (2) of Table 5 present the empirical test results. It can be observed that in the high talent focus group (Tpolicy = 1), the coefficient of De*ΔRev*AQI is not significant, while in the low talent focus group (Tpolicy = 0), the coefficient of De*ΔRev*AQI is significantly negative (p = 0.031, p < 0.05). This indicates that when the government’s focus on talent is high, firms in regions with severe air pollution do not need to offer employees more salary compensation to retain them. The government’s attention to talent can to some extent compensate for the impact of air pollution. The results demonstrate that the positive correlation between air pollution and corporate labor cost stickiness is more pronounced in firms located in areas with low government talent focus.

#### Considering environmental regulation stringency.

Environmental regulation, as a formal institution, may affect the current benefits and future expected benefits of employees. On the one hand, the stringency of environmental regulations represents the intensity of government measures to control pollution and protect the environment. The higher the environmental regulation stringency, the more likely employees in a firm are to have confidence in the future success of environmental governance in that area, and thus may not require as much compensation in their salaries. On the other hand, environmental regulation prompts firms to reduce pollution to meet government requirements [72–74]. Firms in regions with high environmental regulation stringency have a higher demand for high-skilled talents [75,76], while they have a lower demand for ordinary employees and are less likely to take measures to retain them when income declines. Ordinary employees may flow to firms in less polluted areas with less demanding requirements. This paper speculates that the exacerbating effect of air pollution on corporate labor cost stickiness is more pronounced in firms located in areas with low environmental regulation stringency. If the environmental regulation intensity in the city where a firm is located (the proportion of the total investment in urban industrial pollution control to the industrial added value) is higher than the median of all cities in that year, the firm is classified as the strong environmental regulation group (Epolicy = 1); otherwise, it is classified as the weak environmental regulation group (Epolicy = 0). Columns (5) and (6) of Table 5 show the empirical test results. It can be observed that in the strong environmental regulation group (Epolicy = 1), the coefficient of De*ΔRev*AQI is not significant, while in the weak environmental regulation group (Epolicy = 0), the coefficient of De*ΔRev*AQI is significantly negative (p = 0.001, p < 0.01). This indicates that when the environmental regulation stringency is low, firms in regions with severe air pollution need to offer employees more salary compensation. The results demonstrate that the positive correlation between air pollution and corporate labor cost stickiness is more pronounced in firms located in areas with lower environmental regulation stringency.

### Robustness tests

#### Instrumental variable.

This paper follows the approach of Arceo et al (2016) [77], collecting temperature data from the National Aeronautics and Space Administration (NASA). If the natural logarithm of the number of days with temperature inversion in the city where a firm is located, incremented by one, is greater than the median of all firms in the year, Inv is set to 1; otherwise, it is set to 0. De *ΔRev* Inv and De*ΔRev*ESGH*Inv are used as instrumental variables for De*ΔRev*AQI and De*ΔRev* ESGH*AQI, respectively, and two-stage least squares method is used to conduct a robustness test for the main regression. In the troposphere near the Earth’s surface, generally, the temperature at higher altitudes is lower than that at lower altitudes, which makes the air density at higher altitudes higher than that at lower altitudes, facilitating convection to alleviate air pollution. However, under certain conditions such as radiation, advection, or topographical changes, the phenomenon of higher temperatures at higher altitudes than at lower altitudes, known as temperature inversion, can occur. In this case, the air density at higher altitudes is lower than that at lower altitudes, the air layer is more stable, and the convection between upper and lower layers of air is reduced, causing air pollutants to accumulate over a long period and not easily disperse, thus exacerbating air pollution. It is evident that the occurrence of temperature inversion is closely related to the level of air pollution and is not associated with the disturbance terms that affect the explained variable. Accordingly, De*ΔRev*Inv is closely related to De*ΔRev*AQI and is unrelated to the disturbance terms that affect ΔLcost; De*ΔRev*ESGH* Inv is closely related to De*ΔRev*ESGH*AQI and is unrelated to the disturbance terms that affect ΔLcost. Both De*ΔRev*Inv and De*ΔRev*ESGH*Inv meet the requirements for instrumental variables.

Columns (1) and (2) of Table 6 display the first-stage regression results, where the instrumental variable De*ΔRev*Inv has a coefficient of 19.4909, significantly positive (p < 0.01), and De*ΔRev*ESGH*Inv has a coefficient of 19.9274, significantly positive (p < 0.01). The second-stage regression results show that the coefficient of De*ΔRev*AQI is −0.0112, significantly negative (p < 0.01), and the coefficient of De*ΔRev*ESGH*AQI is 0.0127, significantly positive (p < 0.01). The absolute values of these coefficients are greater than those of De*ΔRev*AQI and De*ΔRev*ESGH*AQI in column (3) of Table 3, indicating that the instrumental variables alleviate the problem of coefficient underestimation. The Kleibergen-Paap rk LM value and the Kleibergen-Paap rk Wald F statistic reject the hypotheses of non-identification and weak instrumental variables, indicating that De*ΔRev*Inv and De*ΔRev*ESGH*Inv are good instrumental variables.

**Table 6:** IV regression for air pollution and corporate labor cost stickiness.

Variable	First-stage		Second-stage
	De *ΔRev*AQI	De *ΔRev* ESGH* AQI	ΔLcost
	(1)	(2)	(3)
De *ΔRev* Inv	19.4909***		
	(30.0255)		
De *ΔRev* ESGH* Inv		19.9274***	
		(4.2144)	
De *ΔRev*AQI			−0.0112***
			(−3.4535)
De *ΔRev* ESGH* AQI			0.0127***
			(2.6922)
**ΔRev**	1.1987***	0.4368***	0.6629***
	(5.5489)	(2.9043)	(45.9181)
De *ΔRev	55.7183***	0.1883	−0.0102
	(39.6983)	(0.0560)	(−0.0427)
De *ΔRev* LI	3.6123***	0.2672	−0.1461***
	(11.3537)	(0.2520)	(−6.0550)
De *ΔRev* AI	1.5973***	−0.2677	0.0352*
	(5.8515)	(−0.3541)	(1.6953)
De *ΔRev* Lev	−7.0428***	1.0909	−0.4692***
	(−5.8435)	(0.3924)	(−5.7576)
De *ΔRev* GDPg	972.2469***	−52.9257	−1.4939
	(12.6504)	(−0.2938)	(−0.2620)
De *ΔRev* SucDe	2.0559***	0.7267	0.3508***
	(3.5639)	(0.4187)	(9.0367)
De *ΔRev* ESGH	1.6843***	55.7383***	−1.0417***
	(2.6165)	(21.8761)	(−3.2914)
**Constant**	−1.5523***	−0.7690***	
	(−10.9288)	(−5.9746)	
**Firm/Year**	Yes	Yes	Yes
**Kleibergen-Paap** **rk LM**			30.346
**P-value**			0.000
**Kleibergen-Paap** **rk Wald F statistic**			19.883
**Stock-Yogo bias critical value**			7.03(10%)
**N**	5288	5288	5276
**Within-R** ^**2²**^	0.9205	0.9088	

Note: T-statistics are reported in parentheses. ***, **, and * indicate regression coefficients significant 1%, 5%, and 10%, respectively. If the natural logarithm of the number of days with temperature inversion in the city where a firm is located, incremented by one, is greater than the median of all firms in the year, *Inv* is set to 1; otherwise, it is set to 0.

#### Difference-in-difference analysis.

The CNEPL, which came into effect in 2015, is considered the “strictest Environmental Protection Law (EPL) in Chinese history”. The implementation of the CNEPL has strengthened the government’s environmental regulation, leading to a rapid increase in the number and amount of administrative penalties, mainly targeting polluting firms. This paper conducts a Difference-in-Differences (DID) analysis using the changes in air pollution caused by the implementation of the CNEPL, estimating model (7) with the sample data.


$$\begin{array}{c} \Delta Lcost_{it}=\alpha_{0}+\alpha_{1}\Delta Rev_{it}+(\beta_{0}+\beta_{1}Polluted_{it}*Post_{t}+\beta_{2}AI_{it}+\beta_{3}LI_{it}+\beta_{4}Lev_{it}+\beta_{5}GDPg_{it}\\ +\beta_{6}SucDe_{it}+\beta_{7}ESGH_{it}+\beta_{8}ESGH_{it}*Polluted_{it}*Post_{t})*De_{it}*\Delta Rev_{it}+Firm_{i}+Year_{t}+\varepsilon_{it} \end{array}$$ (7)

In model (7), Polluted is a dummy variable, which takes the value of 1 for firms in heavily polluting industries and 0 otherwise. The variable Post takes the value of 1 for the years 2015 and onwards, and 0 otherwise. The coefficient of Polluted*Post reflects the treatment effect of pollution control on labor cost stickiness. Column (1) of Table 7 shows the results of the DID analysis. The coefficient of Polluted*Post is significantly positive (p < 0.01), indicating that air pollution control has reduced corporate labor cost stickiness. The coefficient of De* ΔRev*ESGH*Polluted*Post is significantly negative (p < 0.01), suggesting that firms with good ESG performance will weaken the negative correlation between air pollution control and corporate labor cost stickiness. The conclusions from the DID analysis are consistent with the main regression results.

**Table 7:** DID analysis, adjustment of control variables and alternative explanatory variable.

Variable	ΔLcost				
	(1)	(2)	(3)	(4)	(5)
**ΔRev**	0.6608***	0.6602***	0.6592***	0.5975***	0.6334***
	(13.1680)	(46.4166)	(46.4330)	(47.8438)	(39.7647)
De *ΔRev	−0.9281***	−0.4165**	−0.6310***	−0.5801***	−0.3221**
	(−3.2675)	(−2.5332)	(−6.8533)	(−6.3698)	(−2.2223)
Polluted*Post	0.3552***				
	(2.5926)				
De *ΔRev*AQI		−0.0026**			
		(−2.0888)			
De *ΔRev*AQI_**t-1**_					−0.0055***
					(−3.6575)
De *ΔRev* PM2.5			−0.0035***		
			(−4.0085)		
De *ΔRev* PM10				−0.0013***	
				(−2.6721)	
De *ΔRev* LI	−0.1803**	−0.1834***	−0.1802***	−0.1288***	−0.1817***
	(−2.1754)	(−8.5234)	(−8.5980)	(−5.5023)	(−8.4424)
De *ΔRev* AI	0.0283	0.0185	−0.0021	0.0010	0.0061
	(0.5331)	(1.0004)	(−0.1173)	(0.0545)	(0.3381)
De *ΔRev* Lev	−0.3189*	−0.4548***	−0.3994***	−0.1931**	−0.4739***
	(−1.9370)	(−5.6832)	(−5.0403)	(−2.2086)	(−5.9379)
De *ΔRev* GDPg	−11.5487	−2.4560	−10.9534**	0.7968	−5.2021
	(−1.5513)	(−0.4520)	(−2.2276)	(0.2005)	(−1.0480)
De *ΔRev* SucDe	0.3273***	0.3556***	0.3433***	0.2195***	0.3505***
	(2.7946)	(9.2138)	(9.0785)	(5.4276)	(8.9742)
De *ΔRev* ESGH	−0.0750	−0.4243***	−0.3461***	−0.3590**	−0.6630***
	(−1.0384)	(−3.4163)	(−5.7567)	(−2.1801)	(−4.5027)
De *ΔRev* ESGH* Polluted*Post	−0.4321***				
	(−2.8219)				
De *ΔRev* ESGH* AQI		0.0035**			
		(1.9929)			
De *ΔRev* ESGH* PM2.5			0.0042***		
			(3.5614)		
De *ΔRev* ESGH* PM10				0.0014**	
				(2.3754)	
De *ΔRev*Growth		−0.9399***			
		(−3.6647)			
De *ΔRev*M2Gr		−1.8374			
		(−1.4229)			
De *ΔRev* ESGH* AQI_**t-1**_					0.0059***
					(2.9528)
**Constant**	0.0653***	0.0662***	0.0672***	0.0576***	
	(5.4660)	(7.0658)	(7.1829)	(6.1239)	
**Firm/Year**	Yes	Yes	Yes	Yes	
**N**	5288	5288	5288	5288	4392
**Within-R** ^**2**^	0.4169	0.4153	0.4149	0.4137	0.4115

Note: T-statistics are reported in parentheses. ***, **, and * indicate regression coefficients significant 1%, 5%, and 10%, respectively. *Polluted* is equal to 1 for the heavy polluting firms and 0 otherwise. *Post* is equal to 1 after 2015 and 0 otherwise. *Growth* is the median growth rate of business income of all firms within the same industry. *M2Gr* is the annual growth rate of the broad money supply M2. *PM2.5* is annual average value of daily PM2.5 in the location of a firm. *PM10* is annual average value of daily PM10 in the location of a firm. *AQI*_t-1_ is the lagged one-period annual average AQI index of the city where a firm is located

#### Adjustment of control variables.

Following the approach of Liu and Liu (2014) [11], This paper adds control variables that represent industry growth potential and monetary policy volatility, namely the median growth rate of business income of all firms within the same industry (Growth) and the annual growth rate of the broad money supply M2 (M2Gr). The regression results in column (2) of Table 7 show that the coefficient of De*ΔRev*AQI is significantly negative (p < 0.05), and the coefficient of De*ΔRev*ESGH*AQI is significantly positive (p < 0.05), indicating that even after adding control variables, the conclusion that air pollution increases corporate labor cost stickiness and that good ESG performance can weaken the positive correlation between air pollution and corporate labor cost stickiness remains unchanged.

#### Alternative explanatory variable.

This paper, following the practices of previous literature, uses the annual average value of daily PM2.5 (PM2.5) and the annual average value of daily PM10 (PM10) in the location of the firm to replace the original explanatory variable AQI as a proxy for the degree of air pollution. The regression results in column (3) of Table 7 show that the coefficient of De*ΔRev*PM2.5 is significantly negative (p < 0.01), and the coefficient of De*ΔRev*ESGH*PM2.5 is significantly positive (p < 0.01). This indicates that air pollution, denoted by the annual average value of daily PM2.5 in the location of a firm, increases the labor cost stickiness, and good ESG performance can mitigate the exacerbating effect of air pollution on corporate labor cost stickiness. The regression results in column (4) of Table 7 show that the coefficient of De*ΔRev*PM10 is significantly negative (p < 0.01), and the coefficient of De*ΔRev*ESGH*PM10 is significantly positive (p < 0.05), indicating that air pollution, denoted by the annual average value of daily PM10 in the location of a firm, increases the labor cost stickiness, and good ESG performance can weaken the positive impact of air pollution on the labor cost stickiness.

This paper uses the lagged one-period annual average AQI index of the city where a firm is located (AQI_t-1_) as a substitute for the current explanatory variable. The regression results, as shown in column (5) of Table 7, indicate that the coefficient of De *ΔRev* AQI_t-1_ remains significantly negative (p < 0.01); the coefficient of De *ΔRev* ESGH* AQI_t-1_ remains significantly positive (p < 0.01), suggesting that air pollution will increase the future corporate labor cost stickiness.

## Conclusions

### Summary of findings

This paper uses sample data from A-share listed firms from 2014 to 2020 in China to conduct an empirical study on the impact and mechanism of air pollution on corporate labor cost stickiness. This study finds that air pollution significantly increases corporate labor cost stickiness (β1 = −0.0031, p = 0.011, i.e., p < 0.05), and this conclusion remains unchanged after addressing endogeneity issues with the instrumental variable method, DID analysis, and other robustness tests. Air pollution increases the stickiness of labor costs for rank-and-file employees in firms (β1 = −0.0051, p = 0.005, i.e., p < 0.01), with no significant impact on the stickiness of labor costs for managers. Corporate good ESG performance can weaken the positive correlation between air pollution and labor cost stickiness. Mechanism tests indicate that the positive correlation between air pollution and labor cost stickiness stems from increased salary compensation and the flow of labor forces. When operating income declines, air pollution may lead firms to offer higher compensation to rank-and-file employees and reduce the loss of employees. This positive correlation is more pronounced in firms located in areas with weaker traditional cultural influence, lower government focus on talent, lower environmental regulation stringency.

### Contributions and implications

This paper enriches the literature on the determinants of corporate labor cost stickiness and contributes to the research on the impact of air pollution on corporate governance. By examining the effect of ecological and environmental quality on corporate labor cost stickiness, this study provides new evidence and insights into the interdependence of environmental and economic benefits. This paper explores the logic behind the changes in labor cost stickiness for different types of human capital within firms. Meanwhile, the analysis of how external culture and policy incentives can mitigate this relationship helps to better understand corporate labor costs comprehensively.

This paper has the following implications:

First, it is essential to implement the concept of sustainable development and strengthen the control of air pollution. The deterioration of the environment can significantly harm economic development, and sacrificing environmental benefits cannot lead to a long-term improvement in economic benefits. Air pollution has no impact on the labor cost stickiness of managers. However, it prompts firms to offer salary compensation to rank-and-file employees to reduce their loss. This compensation reduces corporate cost management efficiency and may harm corporate economic benefits. This paper argues that reducing air pollution is the fundamental solution to the labor issues of firms. Government departments should consistently implement the concept of sustainable development, continue to fight hard against air pollution, and vigorously encourage the development of clean energy projects. They should guide firms to engage in green innovation and sustainable transformation, addressing the root cause of air pollution and promoting high-quality economic development.

Second, it is important to value ESG investment, assessment, and incentives. The research conclusions of this paper indicate that good ESG performance can reduce the positive correlation between air pollution and corporate labor cost stickiness. Improvement in ESG performance can alleviate corporate agency issues and promote the optimal allocation of resources. Therefore, firms should fully consider ESG factors in their strategic planning, actively invest in ESG, to establish a good image in the capital market, gain more support from stakeholders, and promote the enhancement of their long-term value. Governments should pay attention to the assessment and evaluation of corporate ESG, and provide greater policy support for its improvement in ESG performance.

Third, implement inclusive talent and environmental policies to enhance the incentive effects of policies. This paper finds that policy incentives can alleviate the exacerbating effect of air pollution on corporate labor cost stickiness, and the effects of talent and environmental policies can enhance employees’ identification with and positive expectations of the firm and the locality. Implement a more open and inclusive diversified talent policy system, improve talent service mechanisms, and ensure that talents can not only stay but also be effectively utilized. At the same time, vigorously promote the environmental target responsibility system to increase the public’s awareness and participation in environmental protection efforts. This will help achieve a positive situation of “joint construction”, “joint governance”, and “shared benefits”.

### Recommendations for future study

This study mainly focuses on listed firms and differentiates between rank-and-file employees and managers. Future research could expand its scope to include non-listed firms. It could also differentiate the impact on ordinary employees with different skill levels, or investigate how other natural environmental changes affect corporate labor cost stickiness. Such studies may lead to more meaningful conclusions.

## Supporting information

S1 Data (XLS)
